# Supplementary material for: Unveiling the Oxazolidine Character of Pseudoproline Derivatives by Automated Flow Peptide Chemistry
Source: Int J Mol Sci. 2024 Apr 9;25(8):4150. doi: 10.3390/ijms25084150 (PMC11050244; doi:10.3390/ijms25084150)
Supplement: Supplementary file 1 [file ijms-25-04150-s001.zip › ijms-2941062-supplementary.pdf]

Supplementary Materials

Unveiling the Oxazolidine Character of Pseudoproline Derivatives by Automated Flow Peptide Chemistry

Szebasztián Szaniszló [1,2], Antal Csámpai [3], Dániel Horváth [1], Richárd Tomecz [1], Viktor Farkas [4,*] and András Perczel [1,4,*]

[1] Laboratory of Structural Chemistry and Biology, Institute of Chemistry, ELTE Eötvös Loránd University, Pázmány Péter Sétány 1/A, 1117 Budapest, Hungary
[2] ELTE Hevesy György Ph.D. School of Chemistry, ELTE Eötvös Loránd University, Pázmány Péter Sétány 1/A, 1117 Budapest, Hungary
[3] Instutite of Chemistry, ELTE Eötvös Loránd University, Pázmány Péter Sétány 1/A, 1117 Budapest, Hungary
[4] HUN-REN—ELTE Protein Modeling Research Group, ELTE Eötvös Loránd University, Pázmány Péter Sétány 1/A, 1117 Budapest, Hungary
* Correspondence: farkas.viktor@ttk.elte.hu (V.F.); perczel.andras@ttk.elte.hu (A.P.)

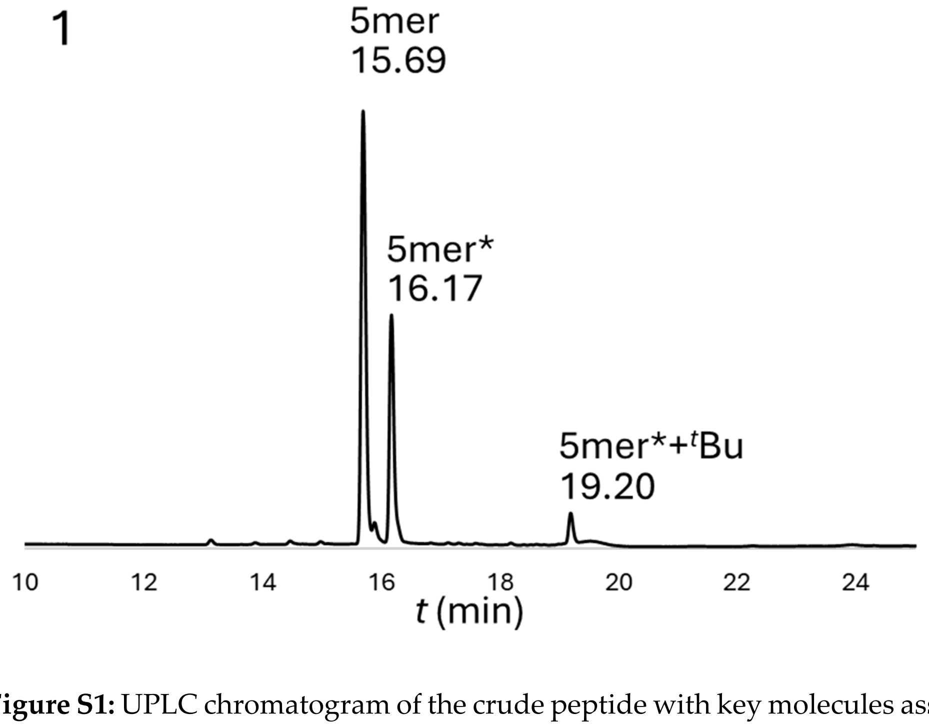

Figure S1: UPLC chromatogram of the crude peptide with key molecules assigned from synthesis #1 (see Table 2 for more details).

Table S1. Calculated and measured monoisotopic masses of molecules from synthesis #1.

Name	Retention time (min)	Calculated monoisotopic mass	Measured monoisotopic mass	Mass error (ppm)
**5mer**	15.69	576.314	576.304	17.352
**5mer***	16.17	616.345	616.338	11.357
**5mer*+ ^{t}Bu**	19.20	672.407	672.287	178.463

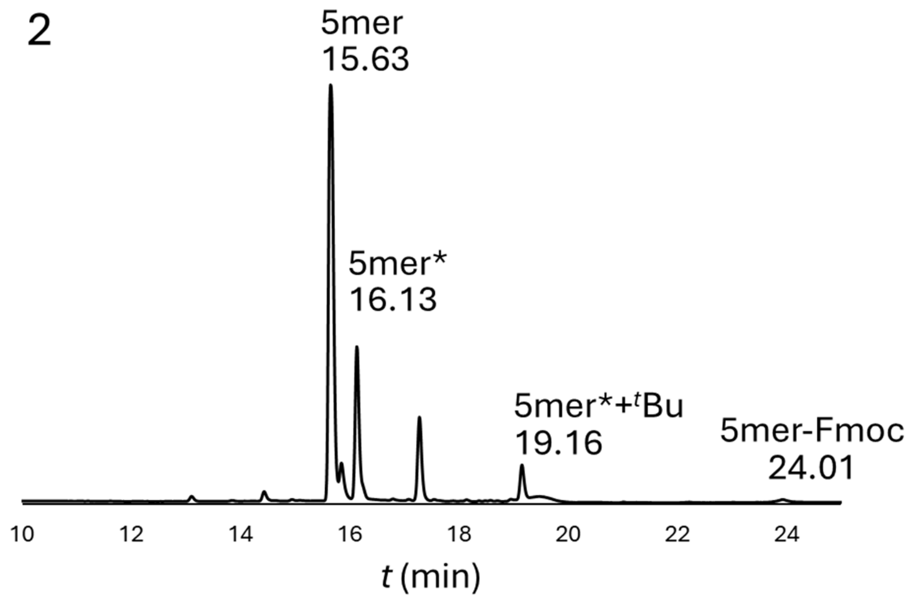

Figure S2: UPLC chromatogram of the crude peptide with key molecules assigned from synthesis #2 (see Table 2 for more details).

Table S2. Calculated and measured monoisotopic masses of molecules from synthesis #2.

Name	Retention time (min)	Calculated monoisotopic mass	Measured monoisotopic mass	Mass error (ppm)
**5mer**	15.63	576.314	576.304	17.352
**5mer***	16.13	616.345	616.338	11.357
**5mer*+ ^{t}Bu**	19.16	672.407	672.287	178.463
**5mer-Fmoc**	24.01	798.382	798.378	5.010

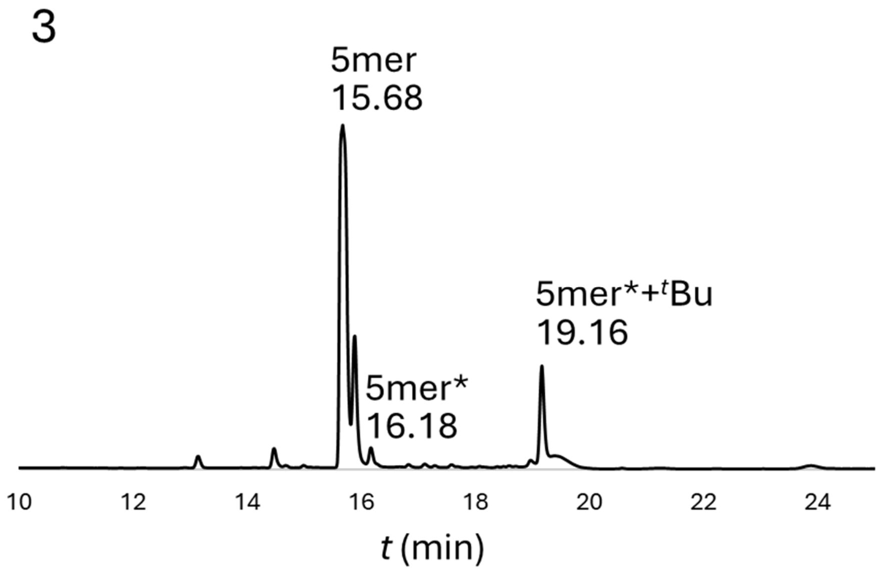

Figure S3: UPLC chromatogram of the crude peptide with key molecules assigned from synthesis #3 (see Table 2 for more details).

Table S3. Calculated and measured monoisotopic masses of molecules from synthesis #3.

Name	Retention time (min)	Calculated monoisotopic mass $[M+H]^+$	Measured monoisotopic mass $[M+H]^+$	Mass error (ppm)
**5mer**	15.68	576.314	576.304	17.352
**5mer***	16.18	616.345	616.320	40.562
**5mer*+^{t}Bu**	19.16	672.407	672.268	206.720

4

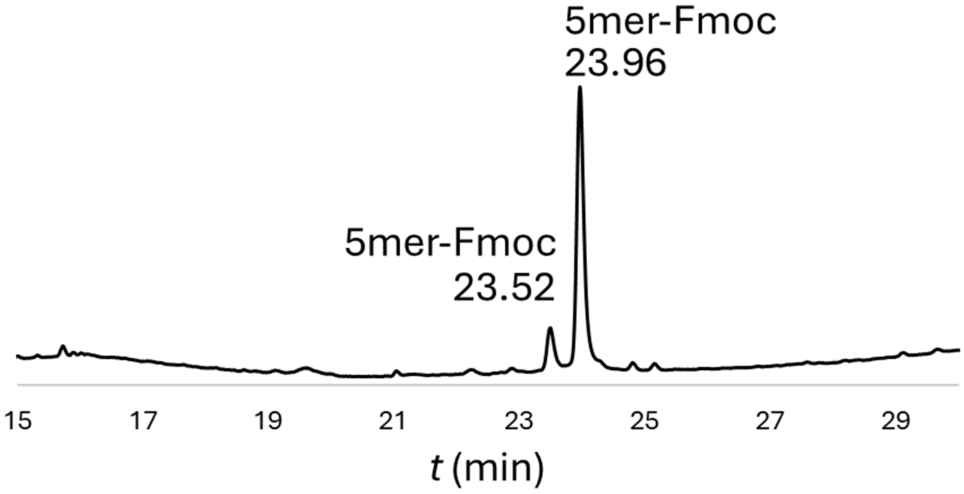

Figure S4: UPLC chromatogram of the crude peptide with key molecules assigned from synthesis #4 (see Table 2 for more details).. The two different peaks refer to the *cis* or *trans* orientation of the Fmoc group.

Table S4. Calculated and measured monoisotopic masses of molecules from synthesis #4.

Name	Retention time (min)	Calculated monoisotopic mass $[M+H]^+$	Measured monoisotopic mass $[M+H]^+$	Mass error (ppm)
**5mer-Fmoc**	23.52	798.382	798.378	5.010
**5mer-Fmoc**	23.96	798.382	798.378	5.010

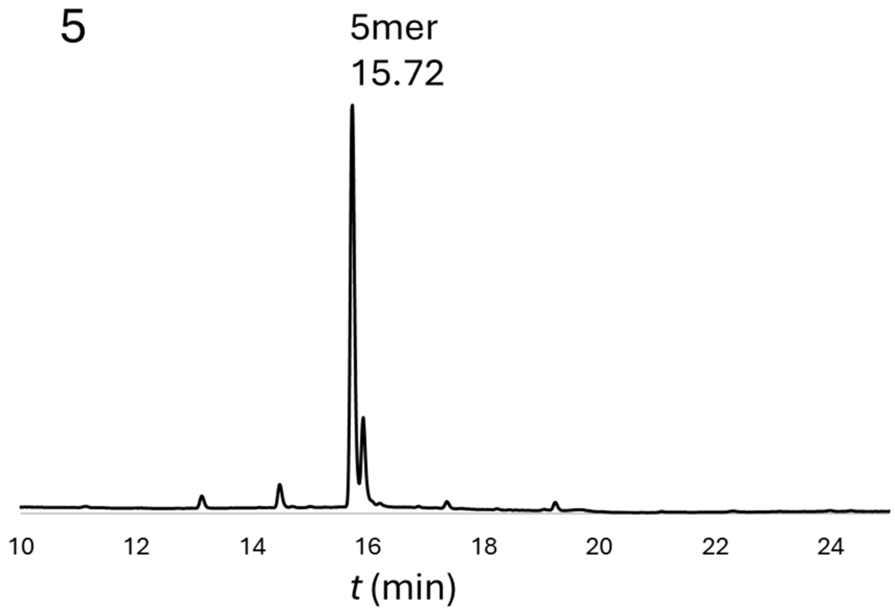

Figure S5: UPLC chromatogram of the crude peptide with key molecules assigned from synthesis #5 (see Table 2 for more details).

Table S5. Calculated and measured monoisotopic masses of molecules from synthesis #5.

Name	Retention time (min)	Calculated monoisotopic mass $[M+H]^+$	Measured monoisotopic mass $[M+H]^+$	Mass error (ppm)
**5mer**	15.72	576.3140	576.375	-105.845

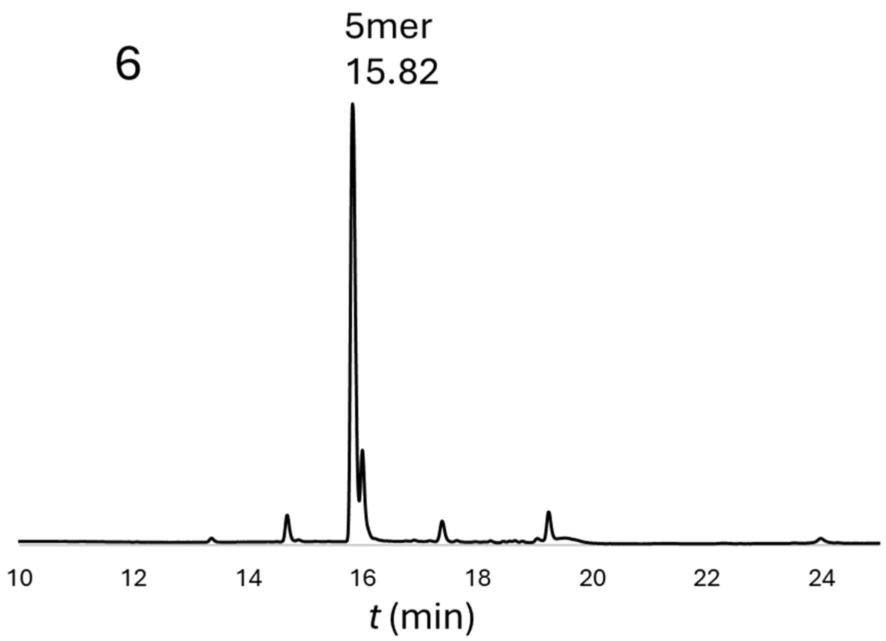

Figure S6: UPLC chromatogram of the crude peptide with key molecules assigned from synthesis #6 (see Table 2 for more details).

Table S6. Calculated and measured monoisotopic masses of molecules from synthesis #6.

Name	Retention time (min)	Calculated monoisotopic mass $[M+H]^+$	Measured monoisotopic mass $[M+H]^+$	Mass error (ppm)
**5mer**	15.82	576.3140	576.393	-137.078

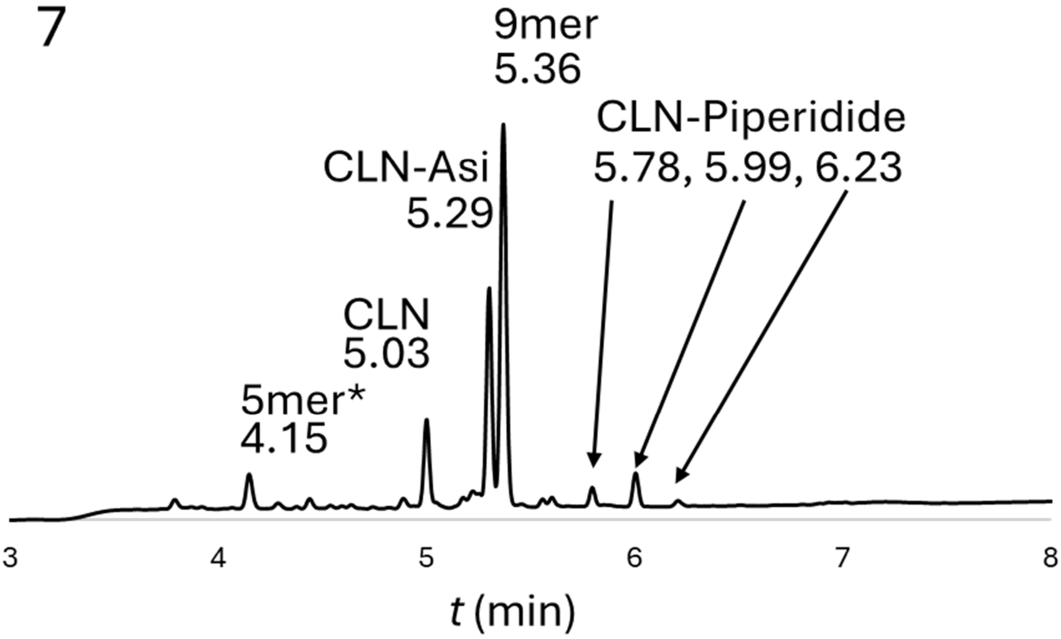

Figure S7: UPLC chromatogram of the crude peptide with key molecules assigned from synthesis #7 (see Table 3 for more details).

Table S7. Calculated and measured monoisotopic masses of molecules from synthesis #7.

Name	Retention time (min)	Calculated monoisotopic mass $[M+H]^+$	Measured monoisotopic mass $[M+H]^+$	Mass error (ppm)
**5mer***	4.15	616.345	616.338	11.357
**CLN**	5.03	1163.573	1163.563	8.594
**CLN-Asi**	5.29	1145.562	1145.552	8.729
**9mer**	5.36	1048.546	1048.538	7.630
**Piperidide**	5.78, 5.99, 6.23	1230.651	1230.640	8.938

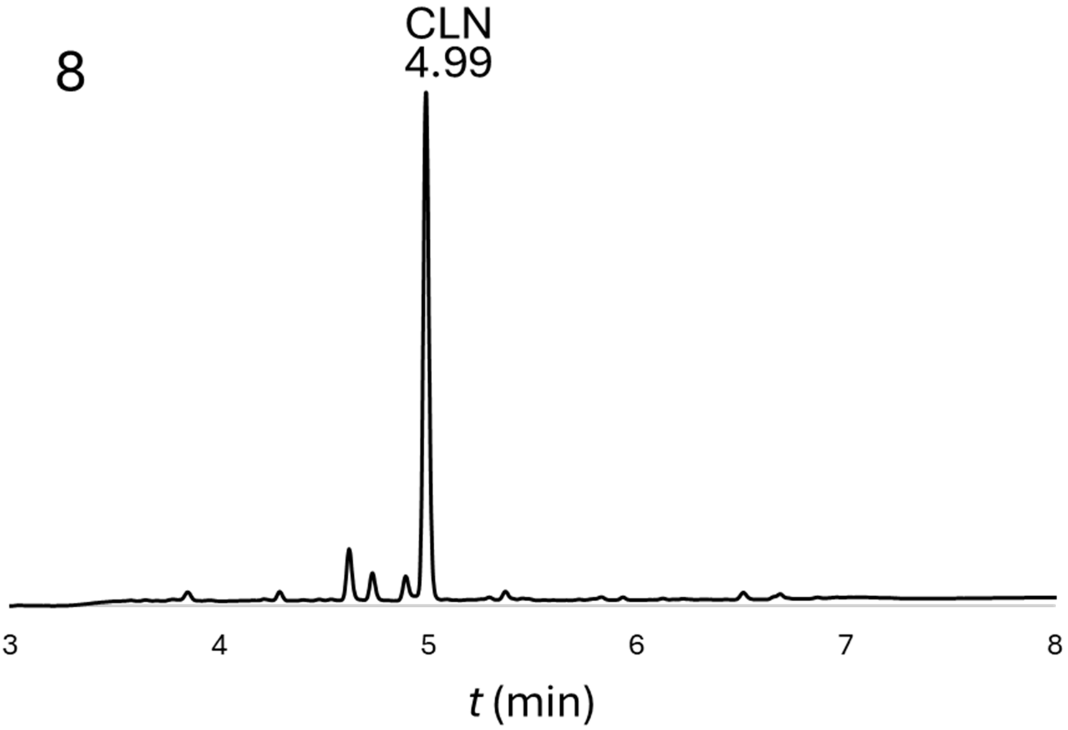

Figure S8: UPLC chromatogram of the crude peptide with key molecules assigned from synthesis #8 (see Table 3 for more details).

Table S8. Calculated and measured monoisotopic masses of molecules from synthesis #8.

Name	Retention time (min)	Calculated monoisotopic mass $[M+H]^+$	Measured monoisotopic mass $[M+H]^+$	Mass error (ppm)
**5mer***	-	616.345	-	-
**CLN**	4.99	1163.573	1163.541	27.501
**CLN-Asi**	-	1145.562	-	-
**9mer**	-	1048.546	-	-
**Piperidide**	-	1230.651	-	-

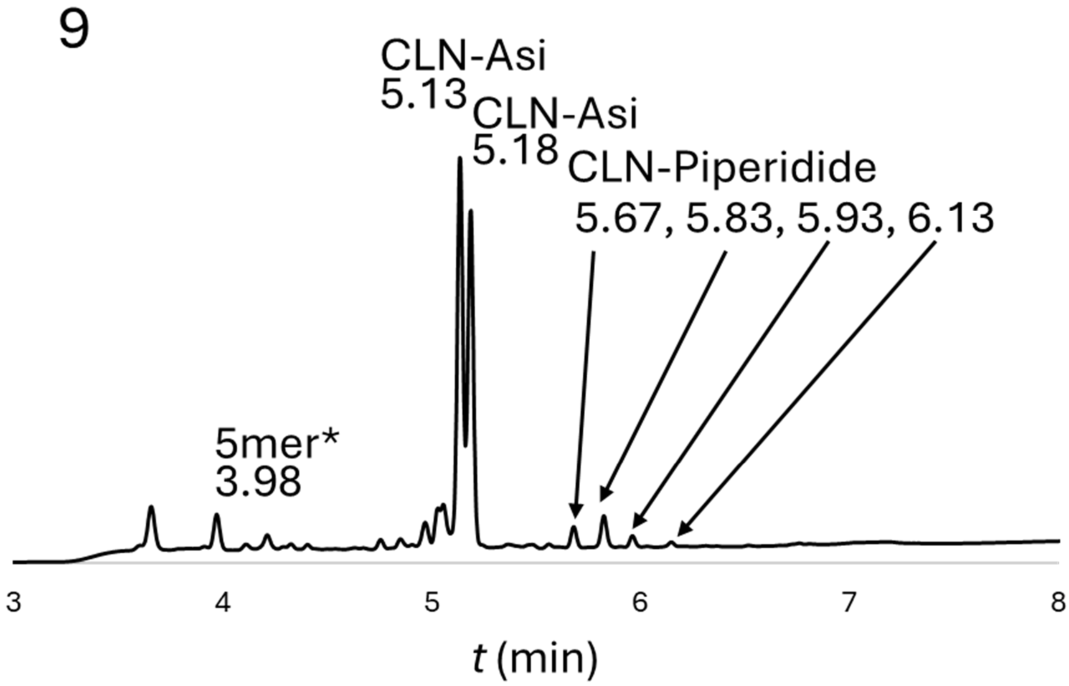

Figure S9: UPLC chromatogram of the crude peptide with key molecules assigned from synthesis #9. In this instance, it is important to note that the peptide contains Ser rather than Thr at the 6th position. (see Table 3 for more details).

Table S9. Calculated and measured monoisotopic masses of molecules from synthesis #9.

Name	Retention time (min)	Calculated monoisotopic mass $[M+H]^+$	Measured monoisotopic mass $[M+H]^+$	Mass error (ppm)
**5mer***	3.98	602.329	602.314	24.903
**CLN**	-	1149.557	-	-
**CLN-Asi**	5.13, 5.18	1131.546	1131.518	24.745
**9mer**	-	1034.530	-	-
**Piperidide**	5.67, 5.83, 5.93, 6.13	1216.636	1216.605	25.480

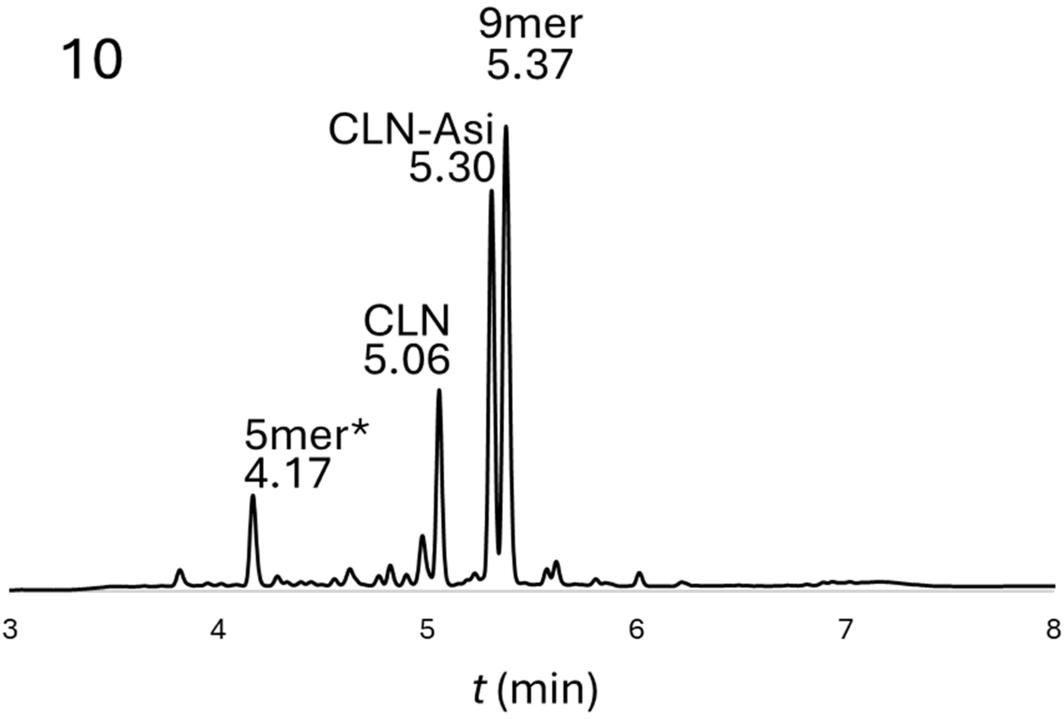

Figure S10: UPLC chromatogram of the crude peptide with key molecules assigned from synthesis #10 (see Table 3 for more details).

Table S10. Calculated and measured monoisotopic masses of molecules from synthesis #10.

Name	Retention time (min)	Calculated monoisotopic mass $[M+H]^+$	Measured monoisotopic mass $[M+H]^+$	Mass error (ppm)
**5mer***	4.17	616.345	616.345	0.000
**CLN**	5.06	1163.573	1163.572	0.859
**CLN-Asi**	5.30	1145.562	1145.566	-3.492
**9mer**	5.37	1048.546	1048.547	-0.954
**Piperidide**		1230.651	-	-

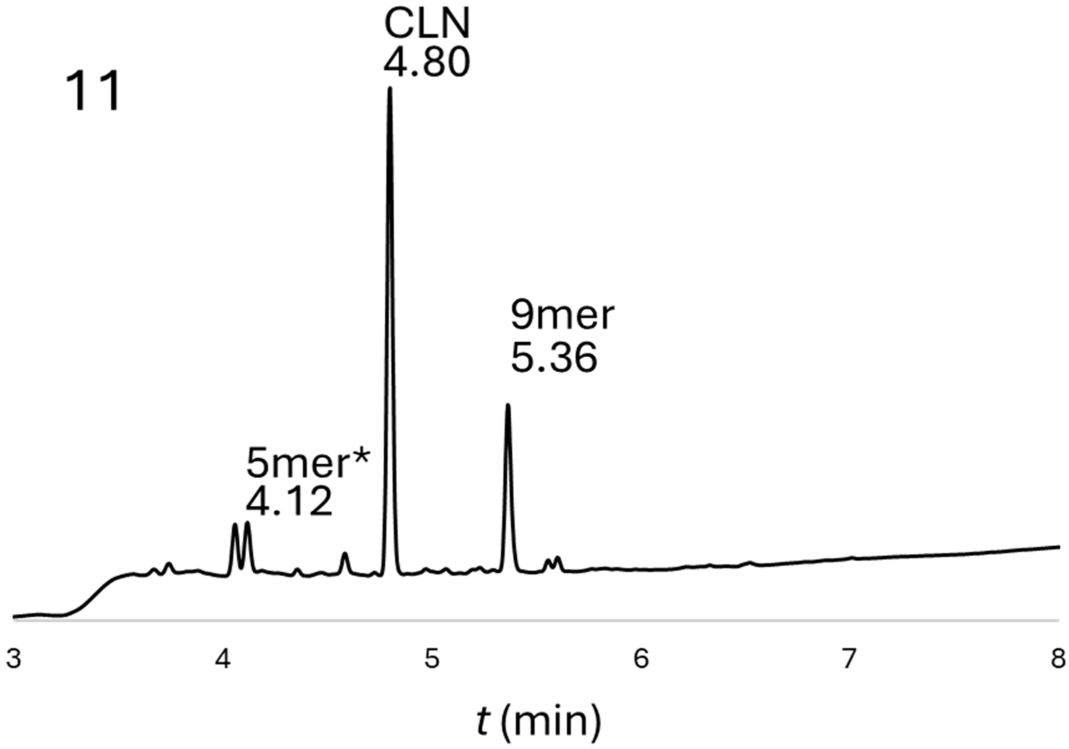

Figure S11: UPLC chromatogram of the crude peptide with key molecules assigned from synthesis #11 (see Table 3 for more details).

Table S11. Calculated and measured monoisotopic masses of molecules from synthesis #11.

Name	Retention time (min)	Calculated monoisotopic mass $[M+H]^+$	Measured monoisotopic mass $[M+H]^+$	Mass error (ppm)
**5mer***	4.12	616.345	616.341	6.490
**CLN**	4.80	1162.589	1162.584	4.301
**CLN-Asi**	-	1144.578	-	-
**9mer**	5.36	1048.546	1048.543	2.861
**Piperidide**	-	1230.651	-	-

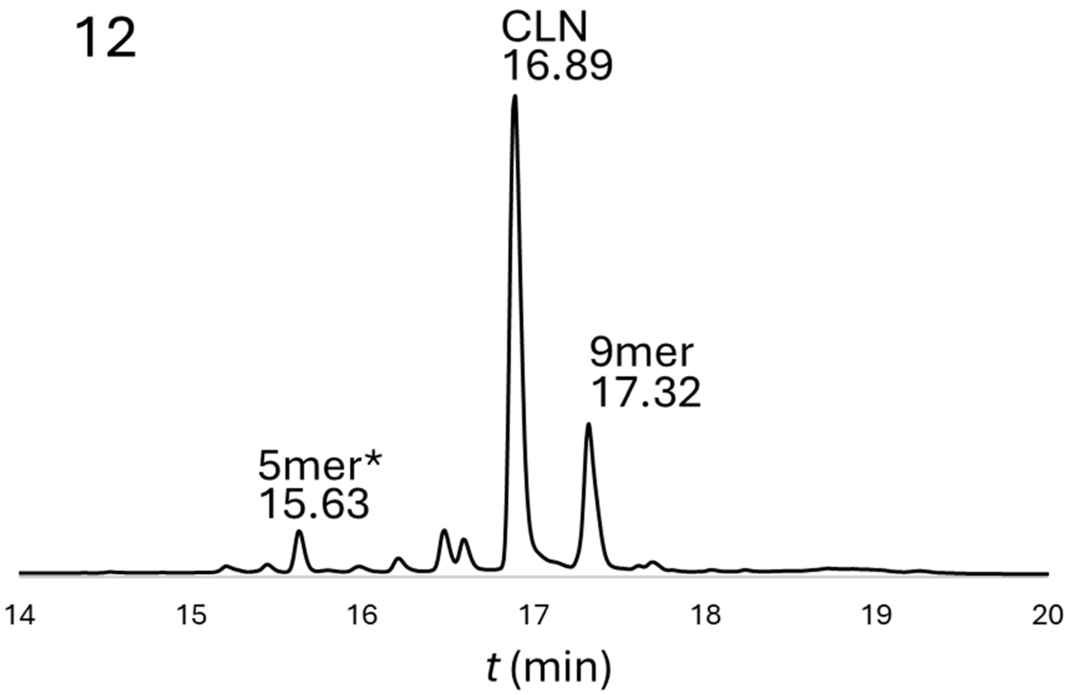

Figure S12: UPLC chromatogram of the crude peptide with key molecules assigned from synthesis #12 (see Table 3 for more details).. Different UPLC gradient was used (molecules are identified with MS)

Table S12. Calculated and measured monoisotopic masses of molecules from synthesis #12.

Name	Retention time (min)	Calculated monoisotopic mass $[M+H]^+$	Measured monoisotopic mass $[M+H]^+$	Mass error (ppm)
**5mer***	15.63	616.345	616.407	-100.593
**CLN**	16.89	1163.573	1163.686	-97.115
**CLN-Asi**	-	1145.562	-	-
**9mer**	17.32	1048.546	1048.658	-106.815
**Piperidide**	-	1230.651	-	-

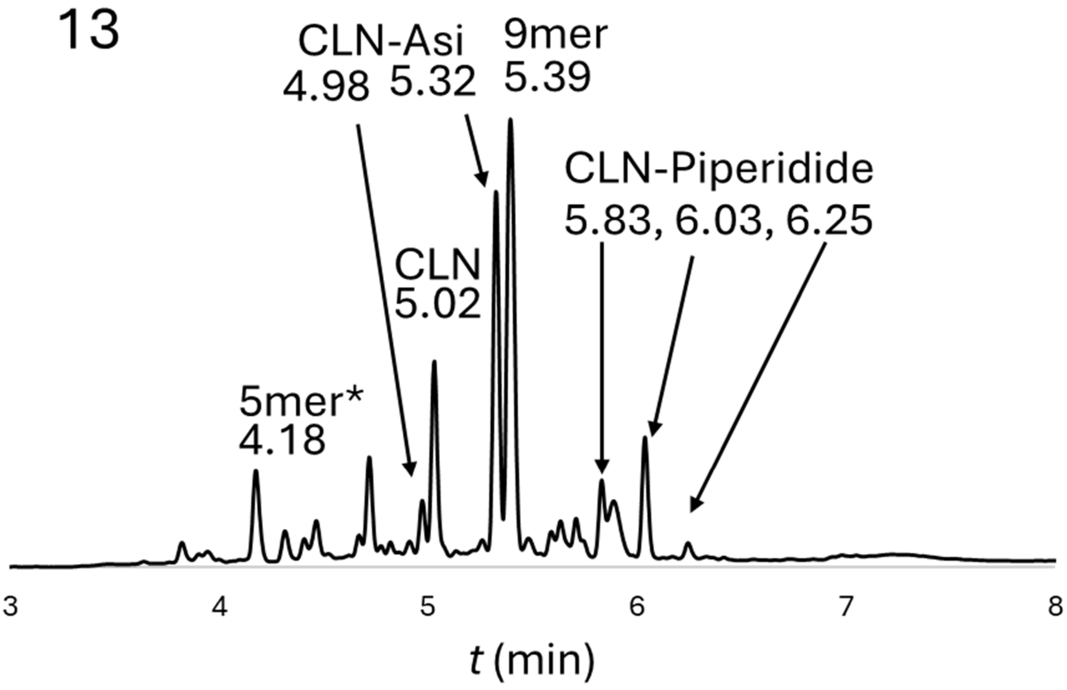

Figure S13: UPLC chromatogram of the crude peptide with key molecules assigned from synthesis #13 (see Table 3 for more details)..

Table S13. Calculated and measured monoisotopic masses of molecules from synthesis #13.

Name	Retention time (min)	Calculated monoisotopic mass [M+H]+	Measured monoisotopic mass [M+H]+	Mass error (ppm)
**5mer***	4.18	616.345	616.351	-9.735
**CLN**	5.02	1163.573	1163.586	-11.172
**CLN-Asi**	4.98, 5.32	1145.562	1145.579	-14.840
**9mer**	5.39	1048.546	1048.560	-13.352
**Piperidide**	5.83, 603, 6.25	1230.651	1230.668	-13.814

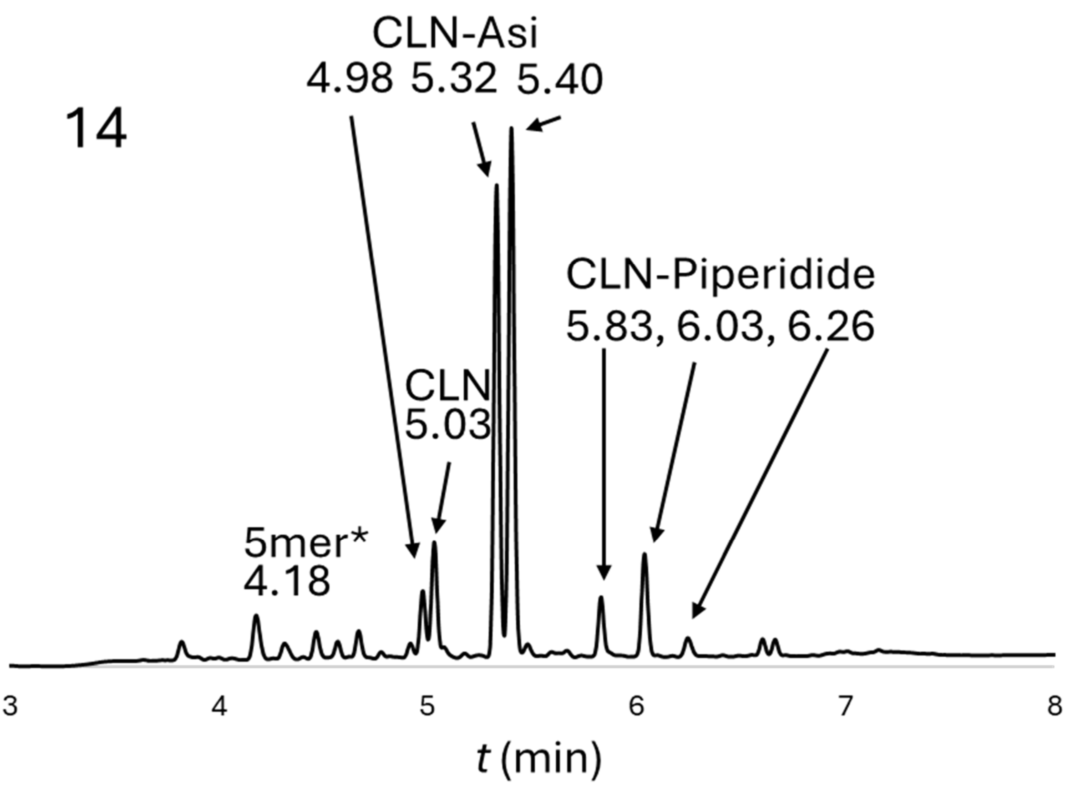

Figure S14: UPLC chromatogram of the crude peptide with key molecules assigned from synthesis #14 (see Table 3 for more details).

Table S14. Calculated and measured monoisotopic masses of molecules from synthesis #14.

Name	Retention time (min)	Calculated monoisotopic mass $[M+H]^+$	Measured monoisotopic mass $[M+H]^+$	Mass error (ppm)
**5mer***	4.18	616.345	616.351	-9.735
**CLN**	5.03	1163.573	1163.591	-15.470
**CLN-Asi**	4.98, 5.32, 5.4	1145.562	1145.579	-14.840
**9mer**	-	1048.546	-	-
**Piperidide**	5.83, 6.03, 6.26	1230.651	1230.668	-13.814

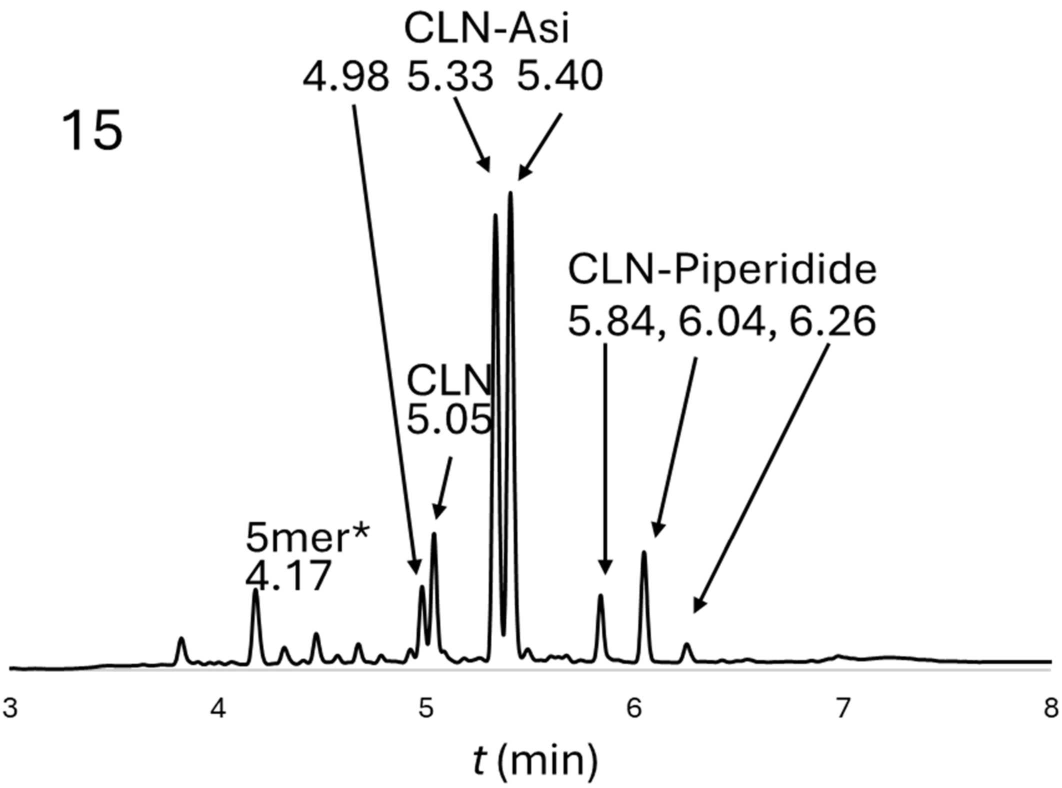

Figure S15: UPLC chromatogram of the crude peptide with key molecules assigned from synthesis #15 (see Table 3 for more details).

Table S15. Calculated and measured monoisotopic masses of molecules from synthesis #15.

Name	Retention time (min)	Calculated monoisotopic mass $[M+H]^+$	Measured monoisotopic mass $[M+H]^+$	Mass error (ppm)
**5mer***	4.17	616.345	616.348	-4.867
**CLN**	5.05	1163.573	1163.582	-7.735
**CLN-Asi**	4.98, 5.33, 5.4	1145.562	1145.570	-6.983
**9mer**	-	1048.546	-	-
**Piperidide**	5.84, 6.04, 6.26	1230.651	1230.664	-10.564

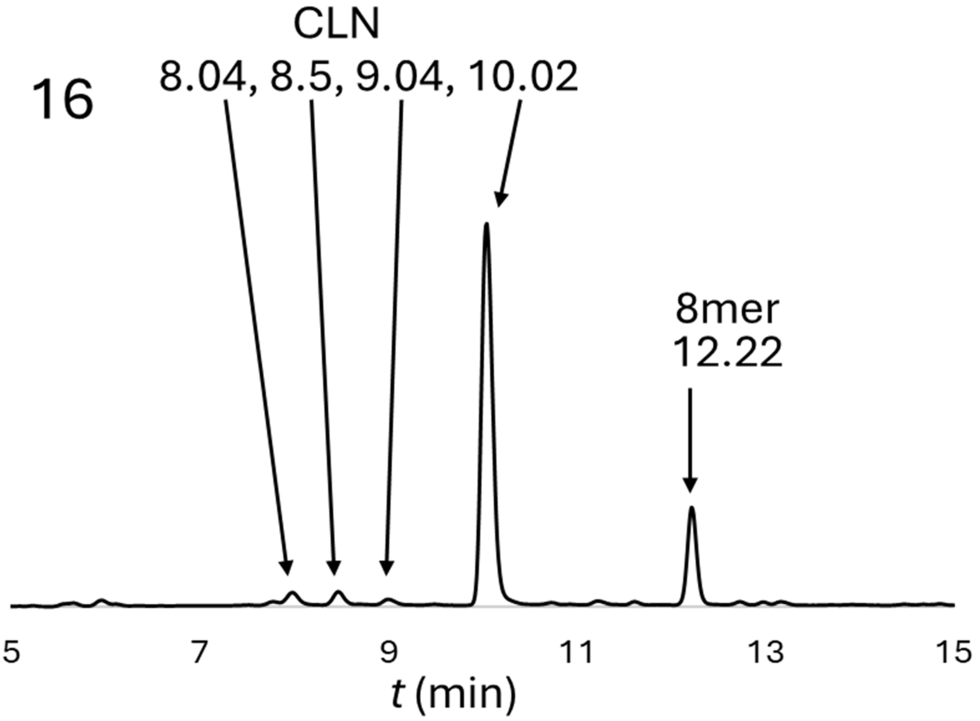

Figure S16: UPLC chromatogram of the crude peptide with key molecules assigned from synthesis #16 (see Table 3 for more details).

Table S16. Calculated and measured monoisotopic masses of molecules from synthesis #16.

Name	Retention time (min)	Calculated monoisotopic mass [M+H]+	Measured monoisotopic mass [M+H]+	Mass error (ppm)
**5mer***	-	616.345	-	-
**CLN**	8.04, 8.5, 9.04, 10.02	1163.573	1163.473	85.942
**CLN-Asi**	-	1145.562	-	-
**9mer**	-	1048.546	-	-
**Piperidide**	-	1230.651	-	-
**8mer**	12.22	947.498	947.417	85.488

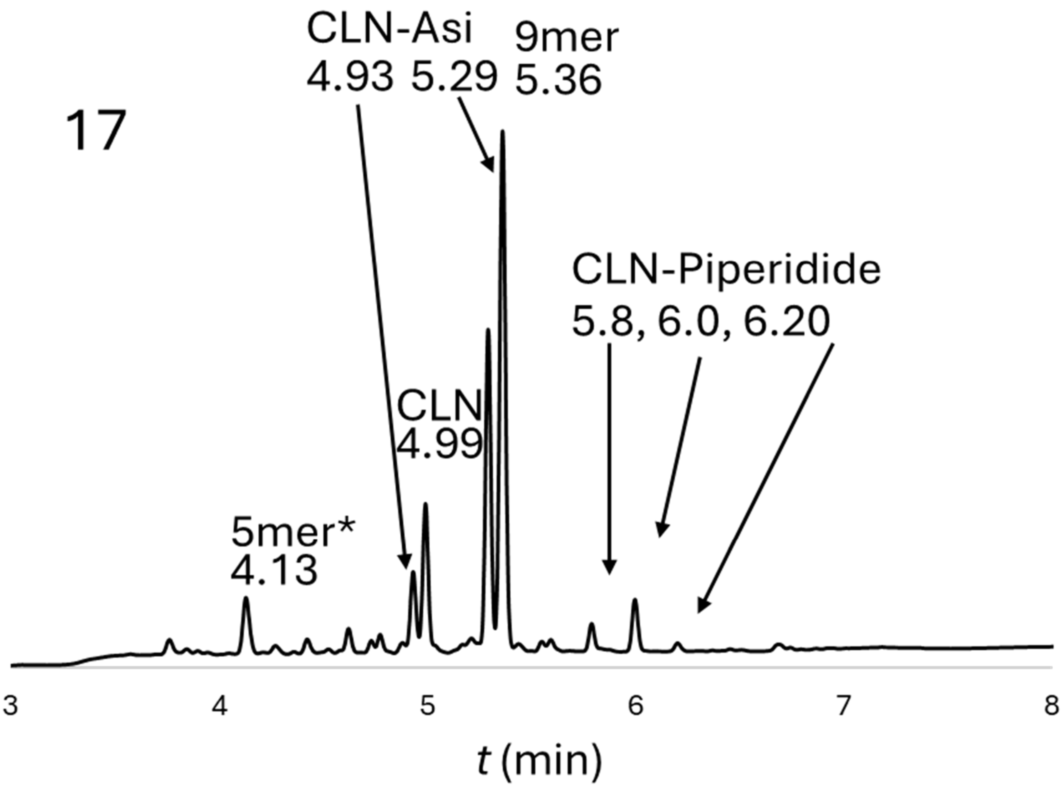

Figure S17: UPLC chromatogram of the crude peptide with key molecules assigned from synthesis #17 (see Table 3 for more details).

Table S17. Calculated and measured monoisotopic masses of molecules from synthesis #17.

Name	Retention time (min)	Calculated monoisotopic mass $[M+H]^+$	Measured monoisotopic mass $[M+H]^+$	Mass error (ppm)
**5mer***	4.13	616.345	616.325	32.449
**CLN**	4.99	1163.573	1163.535	32.658
**CLN-Asi**	4.93, 5.2	1145.562	1145.525	32.299
**9mer**	5.36	1048.546	1048.512	32.426
**Piperidide**	5.8, 6.0, 6.2	1230.651	1230.612	31.691

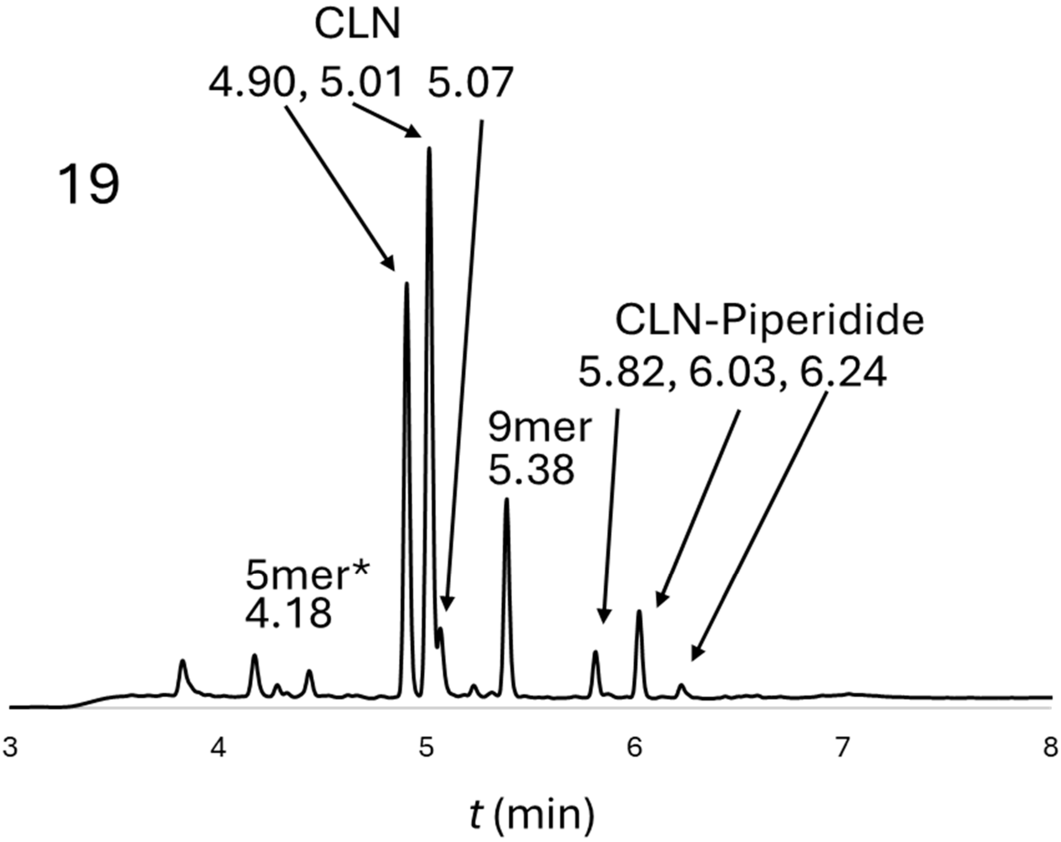

Figure S18: UPLC chromatogram of the crude peptide with key molecules assigned from synthesis #18 (see Table 3 for more details).

Table S18. Calculated and measured monoisotopic masses of molecules from synthesis #18.

Name	Retention time (min)	Calculated monoisotopic mass $[M+H]^+$	Measured monoisotopic mass $[M+H]^+$	
**5mer***	4.18	616.345	616.355	-16.225
**CLN**	4.63, 4,77, 5.02	1163.573	1163.591	-15.470
**CLN-Asi**	4.95, 5.32, 5.39	1145.562	1145.579	-14.840
**9mer**	-	1048.546	-	-
**Piperidide**	5.82, 6.03, 6.24	1230.651	1230.668	-13.814

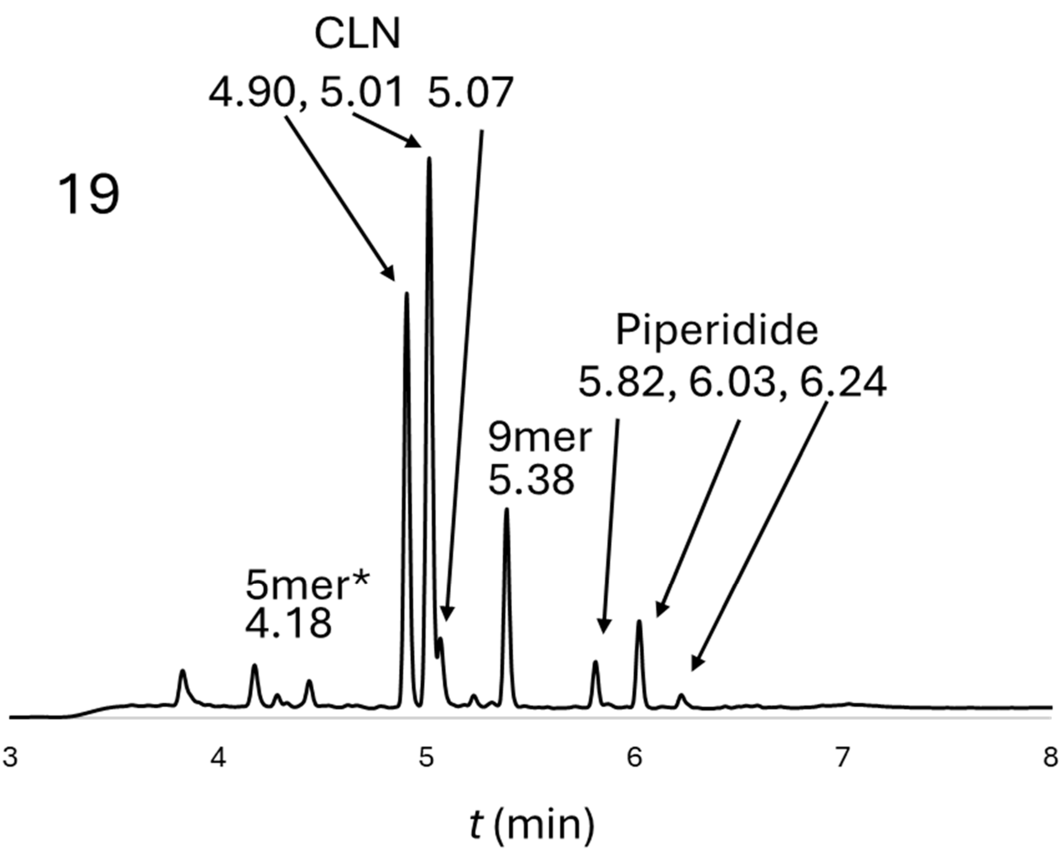

Figure S19: UPLC chromatogram of the crude peptide with key molecules assigned from synthesis #19 (see Table 3 for more details).

Table S19. Calculated and measured monoisotopic masses of molecules from synthesis #19.

Name	Retention time (min)	Calculated monoisotopic mass [M+H]$^+$	Measured monoisotopic mass [M+H]$^+$	Mass error (ppm)
**5mer***	4.18	616.345	616.341	6.490
**CLN**	4.9, 5.01, 5.07	1163.573	1163.568	4.297
**CLN-Asi**	-	1145.562	-	-
**9mer**	5.38	1048.546	1048.54	5.722
**Piperidide**	5.82, 6.03, 6.24	1230.651		-

NMR Study of the CLN and CLN-Asi polypeptides

In **Figure S20 -A**, we can see the amide region of the CLN, where both threonine residues (Thr4 and Thr6) exhibit visible amide proton signals (**Figure S20-A** vertical column around 7.62 ppm, red square) with their respective sequential NOESY cross peaks designating the preceding and subsequent residues. In the suspected aspartimide peptide (Asi), the Thr6 amide proton signal was found to be missing, (**Figure S21-A**) however, the presence of a NOE peak (light green square at column of 8.58 ppm) between Thr6Hα/Hb (5.15 ppm / 5.60 ppm) and Gly7H (8.58 ppm) amide proton indicates the presence of Thr6 side chains. This is only possible if the Thr6 amide proton is missing due to succinimide ring formation of the Asp5 residue, as shown in **Figure S21-A**.

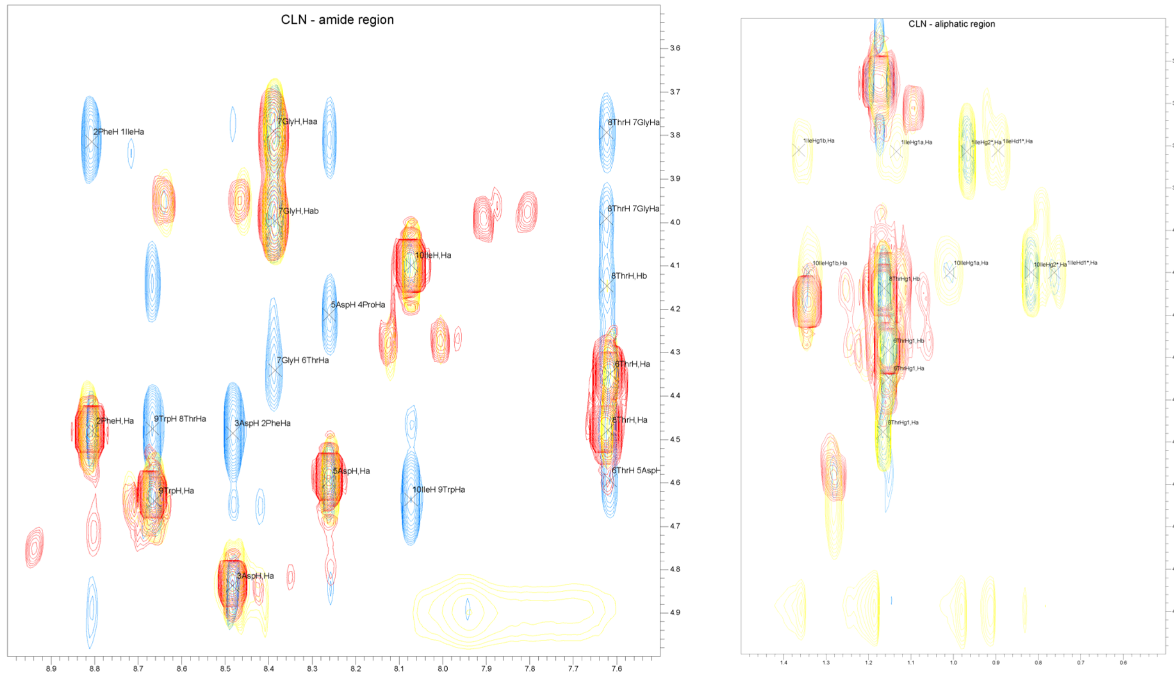

(**A**) (**B**)

Figure S20. (A) The amide region of the CLN proton's NMR resonances: TOCSY/yellow, COSY/red, and NOESY/blue. (B) The side chain region with the proton signals of Thr6 and Thr8 residues marked.

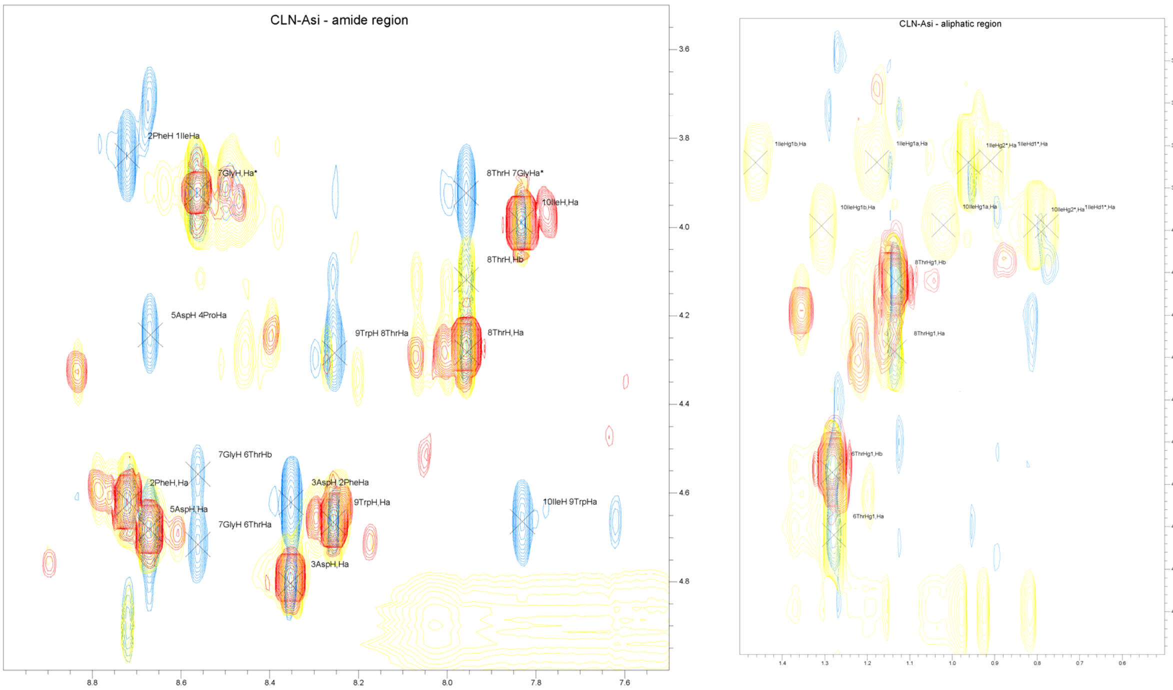

(**A**) (**B**)

Figure S21. (A) The amide region of the CLN-Asi derivative showing the missing Thr6H amide proton signal, however the NOE crosspeak of the Thr6Hα and Gly7H are presented. TOCSY/yellow, COSY/red, and NOESY/blue. (B) Sidechain region with the proton signals of Thr6 and Thr8 residues.

Table S20. NMR shift list of CLN peptide.

		H	HA	HB	HG	HD	HE	HZ
1	Ile	-	3.81	1.95	1.36, 0.97, 1.13	0.9	-	-
2	Phe	8.81	4.48	2.64, 2.74	-	6.88	-	-
3	Asp	8.48	4.84	2.92, 2.45	-	-	-	-
4	Pro	-	4.21	2.32, 2.32	2.00, 2.00	3.81, 3.75	-	-
5	Asp	8.26	4.59	2.83, 2.83	-	-	-	-
6	Thr	7.62	4.35	4.28	1.15	-	-	-
7	Gly	8.39	4.00, 3.79	-	-	-	-	-
8	Thr	7.63	4.48	4.14	1.16	-	-	-
9	Trp	8.66	4.64	3.24, 3.19	-	7.22	10.19, 7.25	
10	Ile	8.07	4.1	1.67	1.34, 1.00, 0.81	0.76	-	-

Table S21. NMR shift list of CLN-Asi peptide.

		H	HA	HB	HG	HD	HE	HZ
1	Ile	-	3.84	1.94	1.47, 1.18, 0.96	0.91	-	-
2	Phe	8.72	4.62	3.09, 2.96	-	7.22	7.48	-
3	Asp	8.35	4.8	2.45, 2.70	-	-	-	-
4	Pro	-	4.24	2.20, 2.20	1.94, 1.94	3.46, 3.72	-	-
5	Asp	8.67	4.68	3.17, 2.80	-	-	-	-
6	Thr	-	4.72	4.56	1.28	-	-	-
7	Gly	8.56	3.92, 3.92	-	-	-	-	-
8	Thr	7.96	4.29	4.12	1.14	-	-	-
9	Trp	8.26	4.66	3.26, 3.26	-	7.21	10.15	7.48
10	Ile	7.83	3.99	1.67	0.80, 1.30, 1.02	0.78	-	-

Table S22. Main parameter sets of the protocols described in Figure 1.

Optimized parameters		protocol s	protocol a	protocol b	protocol c
**Fmoc-Xaa-OH**	eq.	3	3	5	5
**OxymaPure**	eq.	3	3	5	5
**DIC**	eq.	6	6	6	10
**Volume of injection**	mL	1	1	0.8	0.8
**Flow rate Coupling / washing (after Fmoc- deprotection)**	mL/min.	0.3/1	0.3/1	0.3/1	0.15/1
**Cycle time**	min.	7.5	12	12	18
**Temperature**	°C	80	80	80	80
